# Mechanical Enhancement and Water Treatment Efficiency of Nanocomposite PES Membranes: A Study on Akçay Dam Water Filtration Application

**DOI:** 10.1021/acsomega.4c01410

**Published:** 2024-07-15

**Authors:** Sevgi Güneş-Durak, Seren Acarer-Arat, Mertol Tüfekci, İnci Pir, Zeynep Üstkaya, Nurtaç Öz, Neşe Tüfekci

**Affiliations:** † Department of Environmental Engineering, Faculty of Engineering-Architecture, Nevsehir Haci Bektas Veli University, Nevsehir 50300, Turkey; ‡ Department of Environmental Engineering, Faculty of Engineering, Istanbul University−Cerrahpasa, Avcilar, Istanbul 34320, Turkey; § Center for Engineering Research, University of Hertfordshire, Hatfield, Hertfordshire AL10 9AB, United Kingdom; ⊥ School of Physics, Engineering and Computer Science, University of Hertfordshire, Hatfield, Hertfordshire AL10 9AB, United Kingdom; ∥ Faculty of Mechanical Engineering, Istanbul Technical University, Gumussuyu, Istanbul 34437, Turkey; ∇ Department of Water Resources and Treatment Technologies, Sakarya Water and Sewerage Administration (SASKİ), Kudüs Street, Sakarya 54100, Turkey; # Department of Environmental Engineering, Faculty of Engineering, Sakarya University, Esentepe, Sakarya 54187, Turkey

## Abstract

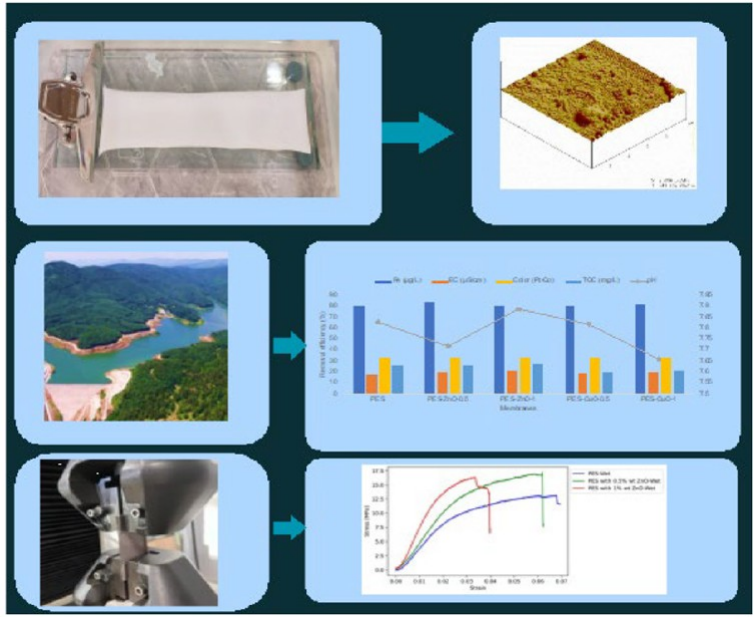

Polymeric membranes are widely used in water treatment because of their ease of fabrication and low cost. The flux and purification performance of membranes can be significantly improved by incorporating appropriate amounts of nanomaterials into the polymeric membrane matrices. In this study, neat poly(ether sulfone) (PES), PES/nano copper oxide (CuO), and PES/nano zinc oxide (ZnO) membranes are fabricated via phase inversion. The pure water flux of the neat PES membrane, which is 355.14 L/m^2^·h, is increased significantly with the addition of nano-CuO and nano-ZnO, and the pure water fluxes of the nanocomposite membranes vary in the range of 392.65–429.74 L/m^2^·h. Moreover, nano CuO and nano ZnO-doped PES nanocomposite membranes exhibit higher conductivity, color, total organic carbon, boron, iron, selenium, barium, and total chromium removal efficiencies than neat PES membranes. The membrane surfaces examined by Scanning Electron Microscopy (SEM) after water filtration revealed that those containing 0.5% wt. nano CuO and nano ZnO are more resistant to fouling than the membrane surfaces containing 1% wt. nano CuO and nano ZnO. Based on the results of this study, 0.5% wt. nano ZnO-doped PES membrane is found to be the most suitable membrane for use in water treatment due to its high pure water flux (427.14 L/m^2^·h), high pollutant removal efficiency, and high fouling resistance. When the mechanical properties of the membranes are examined, the addition of CuO and ZnO nanoparticles increases the membrane stiffness and modulus of elasticity. The addition of 0.5% and 1% for CuO leads to an increase in the modulus of elasticity by 57.95% and 324.43%, respectively, while the addition of 0.5% and 1% for ZnO leads to an increase in the modulus of elasticity by 480.68% and 1802.43%, respectively. At the same time, the tensile strength of the membranes also increases with the addition of nanomaterials.

## 1 Introduction

In recent years, economic development, industrialization, rapid population growth, migration, and climate change have reduced the quantity of water resources and negatively affected water quality.^1^ Only 2.5% of the freshwater in the air, seas, oceans, and underground is frozen in glaciers. The rest are mostly underground, and the portion that can be used as drinking water from water resources worldwide is 0.3% of the total water availability. Although 60% of the human body is water, all living organisms require reliable water.^2^ Moreover, the fact that more than 70% of the world is covered by water remains the same fact that drinking water is scarce in many regions.^3^ Turkey is located in the Mediterranean climate zone, which is affected by global climate change and is among the regions under water stress. It is estimated that Turkey will soon approach the border of countries suffering from “water scarcity” with a decrease in the amount of water per capita per year. Although pollution of water resources is considered an important global problem threatening human health, it is essential to develop advanced, environmentally friendly, and cost-effective technologies for sustainable water treatment.^3^

Membrane filtration systems are good alternatives to conventional systems used in drinking water treatment because of their high separation efficiency, simple operation, low space requirements, and lack of need for chemicals during filtration.^4^ The limitations of water resources and the development of alternative technologies in response to the increase in pollutant parameters have increased the number of studies on nanocomposite membrane applications in removing pollution from drinking water. Nanoparticle-doped composite membranes allow for production and applications that increase membrane functionality, such as resistance to fouling and hydrophilicity. Nanoparticles such as titanium, alumina, silica, silver,^5^ graphene oxide, copper, zinc, titanium dioxide, and some nanofiber structures are preferred as additives to increase flux and hydrophilicity, treatment efficiency, reduce fouling, and improve the thermal and mechanical properties of polymeric membranes.^6^ Polymeric nanocomposite membranes are widely used to improve the properties of standard membranes.^7,8^ They are usually fabricated by blending nanoparticles or fibers through various mechanisms such as mold impregnation (coating) (PC), phase inversion (PI), stretching, interfacial polymerization (IP), sintering, beam etching, and cross-linking (EC).^9^

Poly(ether sulfone) (PES) polymeric materials are preferred because of their suitability for various membrane applications, high performance, and low cost. They have high thermal resistance, can be used over a wide pH range, and have good chemical resistance. Owing to the hydrophobic nature of the PES polymer, the disadvantage is the accumulation and fouling of large molecules on the membrane surface. It is preferred in reverse osmosis (RO), ultrafiltration (UF), and microfiltration (MF) processes because its pore size can be adjusted to the desired size and can be used in tubular and sheet forms.^10^

Although the effect of copper ions added as nanocomposite materials as nanocomposite additives is unknown, copper and copper compounds have been shown to have an effect against different microorganisms, algae, and viruses.^11^ In other words, the effect of copper ions on nanocomposite membranes is limited and more effective in improving antibacterial properties and controlling biofouling.^12^ Zinc oxide (ZnO) is a multifunctional inorganic nanoparticle that has attracted attention because of its catalytic, antibacterial, bactericidal, physical, and chemical properties. In addition, the surface area of ZnO nanoparticles can absorb hydrophilic hydroxyl (OH−) groups, which are higher than those of other inorganic materials.^13^ Reinforcing ZnO inorganic nanoparticles as additives to the membrane improves the hydrophilicity and the mechanical and chemical properties of the polymer.^14,15^ Adding ZnO particles to polymeric membranes increases the resistance to fouling and extends the lifetime of the membranes.^16^ In addition, some studies have shown that the permeability performance of nanocomposite membranes produced by adding ZnO to the polymer is more successful and the surface hydrophilicity is better.^17^

When looking at the phenomena that negatively affect membrane performance in water treatment, biofouling is an important factor that reduces membrane life, and membrane flux and increases energy costs.^18^ For this reason, in recent years, the development of membranes with antibacterial properties to improve membrane performance and working life has been the focus of.^19^ Iron oxide, silver, or copper nanocomposite materials on the surface of membranes can enhance their antibacterial activity. The addition of carbonaceous nanoadditives such as carbon nanotube (CNT), carbon nanofiber (CNF), and graphene oxide (GO) not only improves the mechanical, chemical, and thermal properties but also enhances the water purification performance with fast adsorption kinetics.^20^

The literature indicates a robust exploration into polymer-based membranes’ mechanical, thermal, and antifouling properties enhanced with nanostructures like CuO and ZnO. Zhang et al. (2021) highlight the superhydrophilicity and mechanical durability of CuO microcone decorated membranes, underscoring their potential in oil–water separation.^21^ Nasrollahi et al. (2018) detail how amine-functionalized CuO and ZnO nanoparticles enhance poly(ether sulfone) ultrafiltration membranes’ permeability and antifouling characteristics.^22^ Similarly, Aw et al. (2018) discuss the role of infill density in the tensile and thermoelectric properties of 3D printed composites.^23^ Rajabi et al. (2015) demonstrate that the shape of ZnO nanofillers in PES membranes influences fouling resistance, with nanorods yielding better results.^24^ Parani and Oluwafemi (2020) fabricate superhydrophobic PES-ZnO rod composite membranes, significant for oil–water separation.^25^ Dama et al. (2019) explore the impact of casting speed on the permeability of PES-based membranes.^26^ Zhao et al. (2017) offer insights into the properties of polymer nanocomposites through computer simulations.^27^ Nath and Nilufar (2020) review the additive manufacturing of polymers and composites, indicating the technological evolution in the field.^28^

By changing the nanocomposite material, suitable nanocomposite membranes with different selectivity and permeability that provide very high removal rates can be synthesized to obtain high-quality potable water. In this study, flux tests, filtration tests and SEM, Atomic Force Microscopy (AFM) and mechanical analyzes are carried out to determine the performance, filtration tests and characterization of membranes produced with different properties by adding CuO and ZnO as nanomaterials to poly(ether sulfone) polymer.

## 2 Materials and Methods

### 2.1 Materials

Poly(ether sulfone) (PES) (Veradel 3000P) (average molecular weight: 63 000 g/mol) was used to form the basic structure of the membranes. Dimethyl sulfoxide (DMSO, 99% purity) is obtained from Merck and used as the solvent. Copper oxide (CuO) and zinc oxide (ZnO) are obtained from Nanography to prepare the nanocomposite membranes. The sizes of CuO (99.99% purity) and ZnO (99.5% purity) used in membrane production studies are given as 38 nm and 30–50 nm, respectively.

### 2.2 Membrane Fabrication

**Figure 1:**
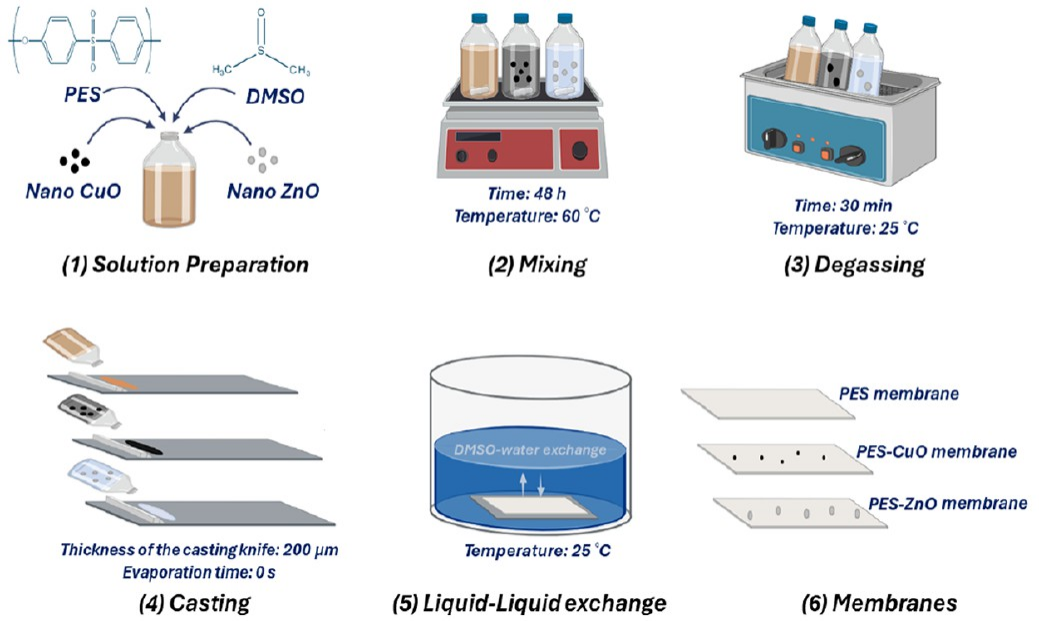
Fabrication of PES, PES-CuO and PES-ZnO membranes by phase inversion method.

Membranes are fabricated by the phase inversion method (Figure 1), which is a widely used method in the production of commercial membranes today. The compositions of the casting solutions used for the fabricated membranes are listed in Table 1. To prepare the PES membrane casting solution, a 16% wt. PES and 84% wt. DMSO is mixed in a capped glass bottle using a heated magnetic stirrer (WiseStir) at 60 °C for 48 h.

**Table 1:** Composition of the Membrane Casting Solutions

Membrane	PES (%)	DMSO (%)	CuO (%)	ZnO (%)
PES	16	84.0	-	-
PES-CuO-0.5	16	83.5	0.5	-
PES-CuO-1	16	83.0	1	-
PES-ZnO-0.5	16	83.5	-	0.5
PES-ZnO-1	16	83.0	-	1

**Figure 2:**
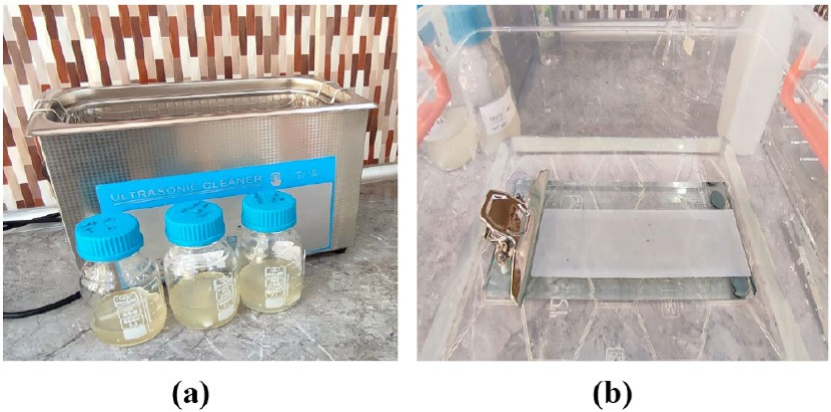
Ultrasonic water bath (a) and phase inversion in the water bath (b).

After obtaining a homogeneous mixture, the solution bottles are placed in an ultrasonic water bath for 30 min at room temperature to remove any bubbles from the solution (Figure 2 (a)). After reaching room temperature, the solutions are spread on a dry flat glass plate with a thickness of 200 μm using a casting knife (TOC Sheen). The glass plate is immersed in a water bath containing distilled water. In the water bath, the membranes are obtained by the phase inversion method as a result of the displacement of DMSO in distilled water and the membrane casting solution (Figure 2 (b)). The obtained membranes are stored in distilled water until further use.

### 2.3 Flux Performance Tests of Membranes

The flux performance tests of the membranes are performed using a vertical flow filtration setup (TIN Engineering). Circular samples are cut from the fabricated membranes to fit inside the filtration cell. After placing the circular membrane samples in the filtration cell, the cell is filled with distilled water. Nitrogen gas is used to provide the pressure required for the filtration. Distilled water is filtered through the membranes in a filtration setup at a pressure of 3 bar, and the permeate is collected in a beaker on a precision balance (AND EJ-610). Time-dependent readings on the precision balance are transferred to a computer, and the fluxes of the membranes are calculated using eq 1.
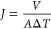
1In eq 1, J, V, A, and Δ*T* represent the flux (L/m^2^·h), volume (L), area (m^2^), and time (h), respectively.

### 2.4 Characterization of Dam Water and Water Treatment Efficiencies of Membranes

In this study, the Akçay Dam water is taken in July 2022, and the water blended with the side streams in the basin is used. In this study, the same procedure applied to pure water flux performance tests is used to filter the blended water, known as Akçay water, through membranes. The Akçay water sample filtered through neat PES and nanocomposite PES membranes is taken using the cold chain method and stored in a clean PET sample bottle in the cold before analysis. Akçay water is characterized in detail, and the characterization results are presented in Table 2.

**Table 2:** Parameters of Akcay Water^a^

Drinking Water Analysis Parameters	RWIHC	Akcay Water (Inlet)
Aluminum (Al) (μg/L)	200	32
Ammonium (NH_4_) (mg/L)	0.5	<0.5
Antimony (Sb) (μg/L)	5	<0.2
Arsenic (As) (μg/L)	10	<1
Copper (Cu) (mg/L)	2	0.02
Barium (Ba) (μg/L)	-	15
Beryllium (Be) (μg/L)	-	<1
Boron (B) (mg/L)	1	0.070
Bromide (Br^–^) (mg/L)	-	<0.2
Turbidity NTU	ACNAC	1.53
Mercury (Hg) (μg/L)	1	<0.1
Zinc (Zn) (mg/L)	-	0.04
Iron (Fe) (μg/L)	200	460
Fluoride (F) (mg/L)	1.5	<0.04
Silver (Ag) (μg/L)	-	<1
Conductivity (μS/cm)	2500	232
Cadmium (Cd) (μg/L)	5	<0.1
Calcium (Ca^2+^) (mg/L)	-	33.14
Chloride (Cl) (mg/L)	250	2.22
Odor (Organoleptic)	ACNAC	Normal
Lead (Pb) (μg/L)	10	0.8
Magnesium (Mg) (mg/L)	-	3.38
Manganese (Mn) (μg/L)	50	40
Nickel (Ni) (μg/L)	20	<3
Nitrate (NO_3_) (mg/L)	50	1.13
Nitrite (NO_2_) (mg/L)	0.5	<0.2
pH	6.5–9.5	7.39
Potassium (K) (mg/L)	-	<1
Color (Pt–Co)	ACNAC	<10
Selenium (Se) (μg/L)	10	<1
Cyanide (μg/L)	50	<20
Sodium (Na) (mg/L)	200	2.03
Sulfate (SO_4_) (mg/L)	250	4.64
Taste (Organoleptic)	ACNAC	Normal
Total Chromium (T-Cr) (μg/L)	50	<1
TOC (mg/L)	NAC	1.85
Total Hardness (CaCO_3_) (°F)	-	9.7

a RWIHC: Regulation on Water Intended for Human Consumption, ACNAC: Acceptable by Consumers and No Abnormal Changes, NAC: No Abnormal Change.

The conductivity, color, TOC, boron, iron, selenium, barium, and total chromium parameters of the permeate of the membranes are analyzed to evaluate the water treatment performance of the membranes. Conductivity is measured using the HQ40d model pH-conductivity device of the HACH company, which measures the hydrogen ion activity in water. The color is determined by the platinum–cobalt stock solution method using a spectrophotometer. TOC analysis is performed by placing the water sample vials filtered through a 0.5 μm cartridge filter into the Sievers brand 5310C model Laboratory TOC Analyzer. To look for boron, iron, selenium, barium and total chromium heavy metals, the analysis sample is first burned with acid to allow the metals to pass into the water in dissolved form, and then placed in tubes and left to be read in the ICP device. The pollutant removal performance of the membranes from water is calculated using eq 2.
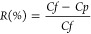
2In eq 2, Cf and Cp correspond to the concentrations in the feed and filtrate streams, respectively.

### 2.5 Surface Characterization of the Membranes

#### 2.5.1 SEM Analysis

Before and after filtration of the Akcay Dam water, the surfaces of the clean and fouled membranes are characterized using a Scanning Electron Microscope (SEM, Philips XL 30 SFEG) at 500x and 2000× magnification. Before SEM analysis, the membranes are dried at room temperature for 1 d, and then the membrane surfaces are coated with gold for 90 s using a coating device.

#### 2.5.2 AFM Analysis

Atomic Force Microscopy (AFM) allows surfaces to be imaged in high resolution and three-dimensional as a result of the interaction of the needle tip structure with the sample. AFM images are taken with the Digital Instruments atomic force microscope device to obtain the roughness (indentations, etc.) and surface topography of all fabricated membranes.

### 2.6 Mechanical Tests of the Membranes

The evaluation of mechanical properties in materials is crucially conducted through the tensile testing method, a standardized approach. This method allows for the quantification of the interaction between applied force and the resultant displacement in materials. Through this experimental procedure, essential data is gathered, enabling the construction of a stress–strain graph. From this, it is possible to ascertain key mechanical properties such as the modulus of elasticity, the tensile strength, and the elongation at break. These properties are important for a comprehensive understanding of a material’s mechanical behavior, and they provide a basis for a quantitative comparison with other samples. In this study, the strain rate for the quasi-static evaluations is established at a consistent 1% strain per minute. To comprehensively assess the material properties, each membrane configuration undergoes testing under both hydrated and dehydrated states (subjected to air-drying for 24 h under standard environmental conditions). This approach aims to determine the effect of moisture on the mechanical properties of membranes. Aluminum fixtures are employed at the ends of each sample to avoid slipping from the clamps during the tests. The tensile tests were conducted using the Shimadzu AG-IS 50kN universal testing machine.

### 2.7 Porosity of Membranes

The porosity of the membranes is determined by the gravimetric method using eq 3. To determine the wet and dry weight of the membranes, the membrane samples are placed in aluminum weighing containers and then dried in a 45 °C oven (Nuve EN 500) for 45 h. The weights of the dried membranes are measured with a precision scale (Precisa XB 220A). After the dry membrane samples are immersed in distilled water for 2 min, the water on them is immediately removed with a blotting paper and the wet weight of the membranes is measured with a precision balance.
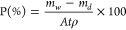
3In eq 3, P represents the porosity of the membrane. m_*w*_ and m_*d*_ represent the wet and dry weights of the membrane (g). A represents the membrane area (cm^2^). t represents the membrane thickness (cm), and ρ represents the density of water (0.998 g/cm^3^).

## 3 Results and Discussion

### 3.1 Membrane Flux Performance

**Figure 3:**
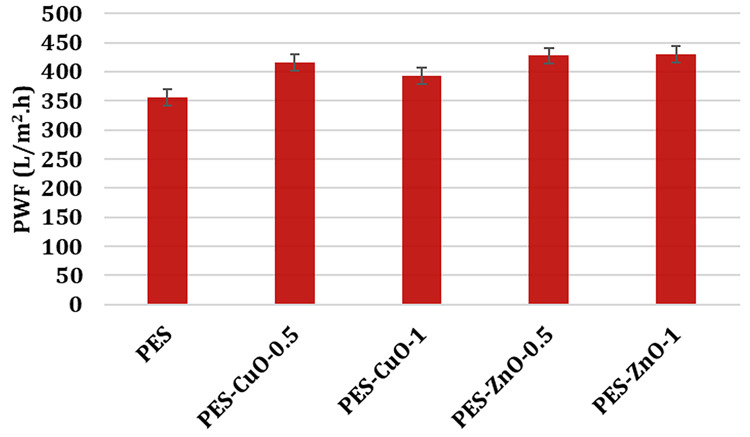
Pure water flux of the membranes.

Flux is an indicator of the water treated per unit of time. A higher flux means that more water becomes potable or usable within a given time. Figure 3 shows the pure water flux of the membranes produced at a pressure of 3 bar. Among the membranes produced, the lowest flux is found for the PES membrane at 355.14 L/m^2^·h. The flux performance of all nanocomposite membranes fabricated using nano CuO and nano ZnO is higher than that of the neat PES membrane. The incorporation of hydrophilic materials, especially nano CuO and nano ZnO, into the relatively hydrophobic PES membrane matrix increases the surface hydrophilicity of the membrane.^29,30^ Nano CuO and nano ZnO on the membrane surface can help water to pass through the membrane more easily by allowing the water filtered through the membrane to be more attracted to the surface and spread more easily on the membrane surface. Second, hydrophilic nanomaterials absorb water and allow it to pass through the membrane faster. For these reasons, the flux of the PES membrane increases to 415.27 and 392.65 L/m^2^·h with the addition of 0.5% wt. and 1% wt. CuO, respectively, while it increases to 427.14 and 429.74 L/m^2^·h with the addition of 0.5% wt. and 1% wt. ZnO. Because, ZnO has a smaller pore size than CuO, which facilitates the passage of water molecules through the membrane. Additionally, ZnO provides a larger surface area, allowing for greater contact between water molecules and the membrane, resulting in more efficient water treatment. Furthermore, ZnO is more hydrophilic than CuO, further enhancing the ease of water molecule passage through the membrane.^31^ Moreover, the porosity value of the PES membrane is 55.62%, while the porosities of the PES-CuO-0.5 and PES-ZnO-0.5 membranes are calculated as 48.34% and 58.82%, respectively. Water filtration may have been facilitated more through nano ZnO-doped PES membrane due to both the more hydrophilic property of nano ZnO compared to nano CuO and its contribution to increasing membrane porosity.

When 0.5% wt. nano CuO is added to the PES membrane, the flux performance increases by 16.9%, while the flux performance increases by 10.5% when 1% wt. nano CuO is added. The decrease in flux with increasing amounts of nano CuO in the PES membrane indicates that high amounts of nano CuO cannot be homogeneously dispersed in the polymer matrix. As the amount of nanomaterial added to the membrane casting solution is increased, the viscosity of the solution also increases.^32^ Adding high amounts of nano CuO to the PES membrane, that is, 1% wt. nano CuO, causes the viscosity of the membrane casting solution to increase significantly, so the nano CuO is not very well dispersed in the casting solution. Failure to achieve a good dispersion in the membrane matrix leads to performance degradation.

Addition of 0.5% wt. and 1% wt. ZnO on the PES membrane increases the membrane’s flux by 20.2% and 21.0%, respectively. Nano ZnO performs better than nano CuO in increasing the flux performance of the PES membrane. Moreover, even when the amount of nano ZnO in the membrane matrix is high, that is, 1% wt. nano ZnO, no decrease in flux performance is detected. This indicates that nano ZnO can be better dispersed in the PES membrane matrix than the same amount of nano CuO.

### 3.2 Treatment Performance of Membranes

**Figure 4:**
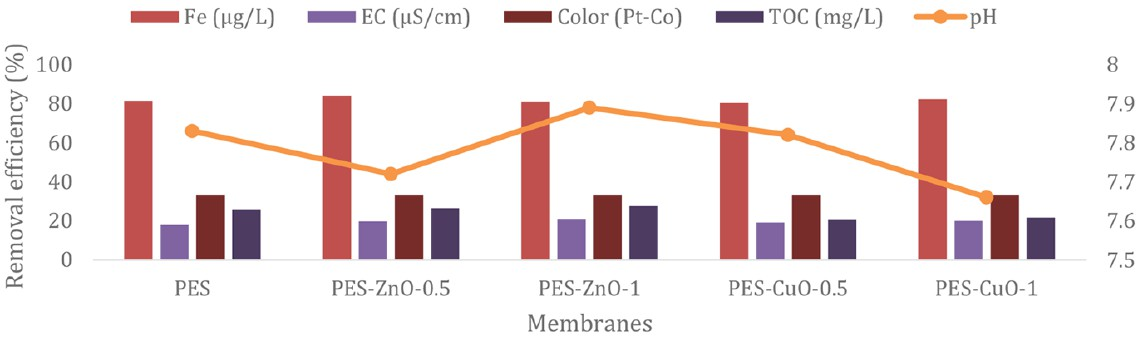
Removal efficiency of indicator parameters after fabricated membranes filtration.

High flux and separation performance are required for water treatment membranes. Membranes with a high separation performance effectively remove pollutants from water and provide more reliable water. Figure 4 shows removal efficiency of indicator parameters of neat and nanocomposite PES membranes. Among all the membranes, the neat PES membrane exhibits the lowest removal efficiency for organic and inorganic pollutants. The increased pollutant removal efficiency with the addition of nano CuO and nano ZnO to the neat PES membrane is thought to be due to the high viscosity of the nanomaterial-doped membrane casting solutions, resulting in denser membranes. In general, PES/ZnO membranes have higher removal efficiencies than those of PES/CuO membranes. Among all the membranes, the PES-ZnO-0.5 membrane exhibits the best performance for the removal of organic and inorganic pollutants from water. The fact that nanocomposite membranes, especially PES-ZnO-0.5, have higher pollutant removal efficiency than neat PES membrane demonstrates that nanocomposite membranes are more successful in water treatment applications. Therefore, nano CuO and nano ZnO-doped PES membranes produced in this study can be used in water treatment to obtain more reliable water or to remove more pollutants from the water as a pretreatment before other membrane processes.

**Figure 5:**
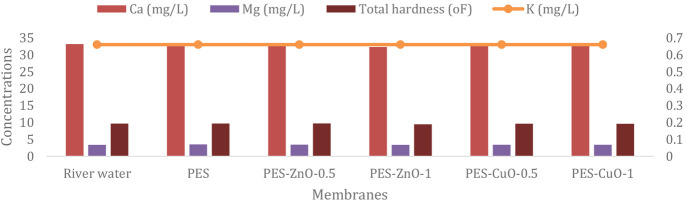
Concentration changes of optional parameters after fabricated membranes filtration.

**Figure 6:**
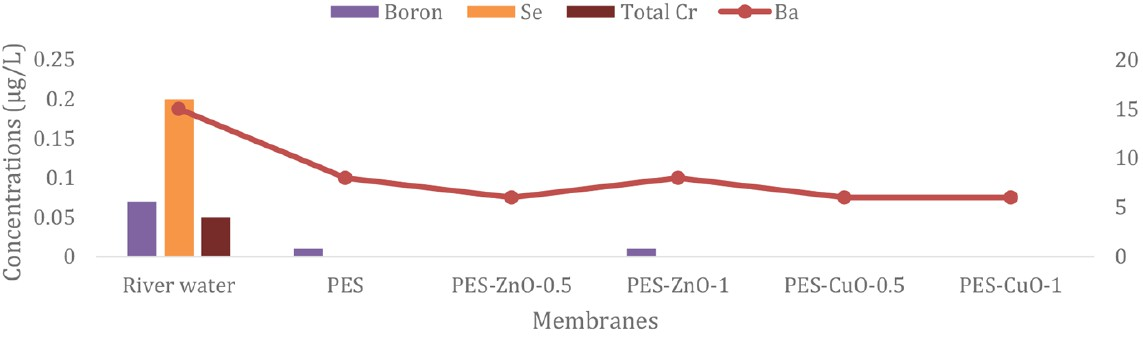
Concentration changes of chemical parameters after fabricated membranes filtration.

Figure 5 shows no significant change in concentration in the optional parameters such as Ca, Mg, total hardness, and K after filtration for all membranes fabricated. Figure 6 shows the performance of the membranes produced after filtration for the removal of chemical pollutants in river water. All the studied parameters are removed at significant rates. All membranes completely remove Se and total chromium. It is possible to say that pure PES and composite PES membranes effectively treat chemical removal. In membrane separation processes, membrane surface charge plays a vital role in filtration. PES membrane is negatively loaded without Zn O and CuO addition. The pollutant parameters of the PES membrane enriched with ZnO and CuO, i.e. cation removal, result from ion exchange with the negatively charged surface of the PES membrane. However, no clear information in the literature shows that PES membranes are effective for anion or cation removal.

### 3.3 Surface Images of the Clean and Fouled Membranes

**Figure 7:**
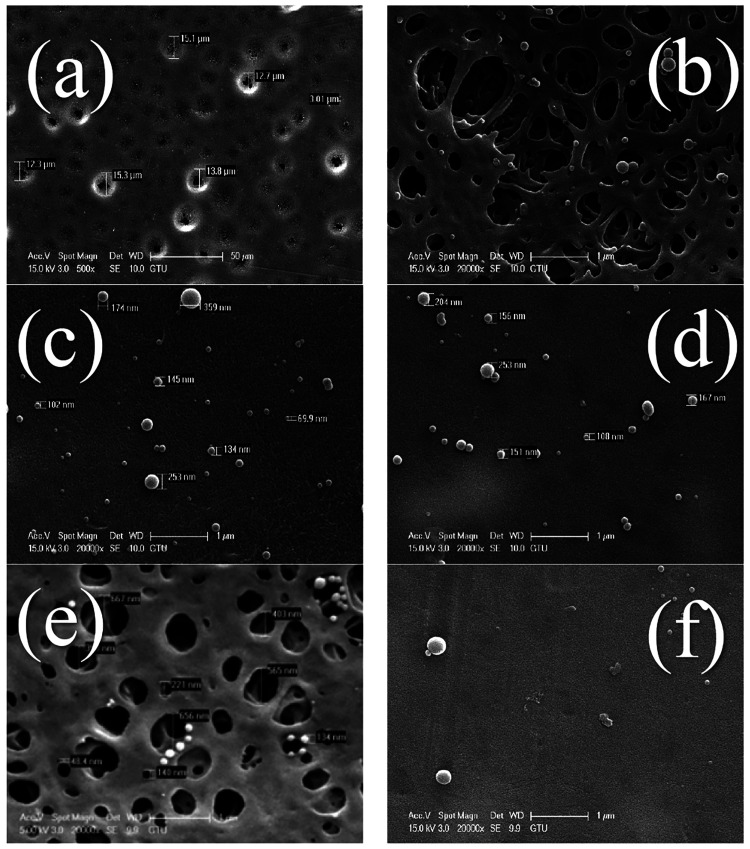
Surface images of the membranes before filtration: (a, b) PES, (c) PES-CuO-0.5, (d) PES-CuO-1, (e) PES-ZnO-0.5, (f) PES-ZnO-1.

Figure 7 (a), (b), (c), (d), (e), and (f) show SEM images of neat PES and PES-CuO-0.5, PES-CuO-1, PES-ZnO-0.5, PES-ZnO-1 composite membranes, respectively. When the images are examined, it is observed that all membranes showed asymmetric structures. Microporous top surfaces show large voids and finger-like structures in an asymmetric and highly inhomogeneous structure. It is possible to say that the average pore diameter of the neat PES membrane is 9 μm, the PES-CuO-0.5 membrane is 215 nm, the PES-CuO-1 membrane is 181 nm, the PES-ZnO-0.5 membrane is 355 nm and PES-ZnO-1 membrane is 191 nm. When nanoparticles are added to the neat polymer, a decrease of up to 97.9% in pore diameter occurs. However, the increase in the nanomaterial concentration in the membrane content decreased the pore diameter. In addition, in the SEM images before filtration, it is seen that the pore sizes of the ZnO membrane are larger than the pore sizes of the CuO membrane. ZnO nanoparticles tend to precipitate more than CuO nanoparticles, leading to the clogging of membrane pores and the formation of larger pores. Additionally, the preparation conditions of the membrane, such as mixture homogeneity, temperature, pH, and pore size, may also contribute to this phenomenon.

The surface SEM images of the membranes show a dense porous structure at the top. The main factor determining the surface morphological properties of the membranes synthesized in the phase transformation method by immersion precipitation is the rate and rate of exchange between the solvent and nonsolvent phase. If this phase transformation between solvent and nonsolvent occurs quickly and with a high exchange rate, the pore sizes obtained in the membrane are large, and the porosity is high.^33^ The slower phase transformation between solvent and solvent leads to smaller pore sizes and the number of pores in the membrane. The polymeric membrane solution’s viscosity determines the solvent–nonsolvent transition’s speed. As the solution viscosity increases, the phase change between solvent and solvent and the formation rate of the membrane slows down.^34^

**Figure 8:**
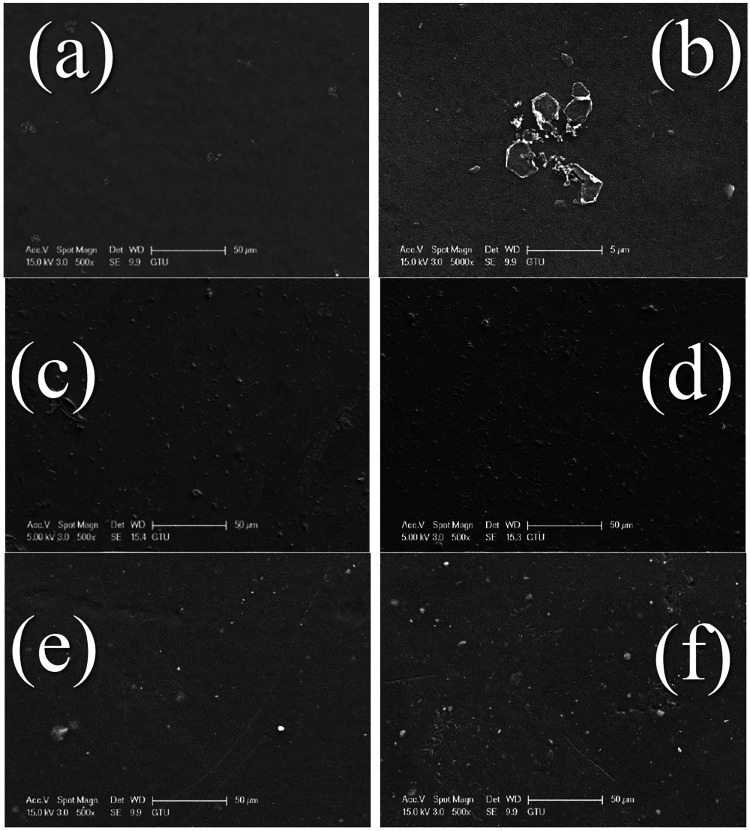
Surface images of fouled membranes after filtration: (a, b) PES, (c) PES-CuO-0.5, (d) PES-CuO-1, (e) PES-ZnO-0.5, (f) PES-ZnO-1.

During water treatment with membranes, contaminants accumulate on the membrane surface and pores. In addition to causing flux reduction during filtration, the contaminants accumulated in the membrane degrade the structure of the membranes and shorten the membrane life. In addition, membranes with low fouling resistance require additional cleaning or replacement, which increases the operating costs of such membranes. After filtration, the surfaces of the membranes are characterized by SEM, as shown in Figure 8. The membranes exhibit dense and rough surfaces due to the accumulation of contaminants in water on the membrane surface and pores.

The nano CuO-doped nanocomposite PES membranes exhibit a rough surface with more contaminants on the surface compared to the neat PES membrane. The surface of the PES/ZnO nanocomposite membranes accumulate fewer contaminants than that of the PES/CuO nanocomposite membranes. The results obtained from SEM images reveal that nano ZnO is more effective than nano CuO in improving the fouling resistance of the PES membrane. For both nanomaterials, it is observed that increasing the ratio from 0.5% to 1% resulting in more membrane fouling. This may be attributed to the fact that higher amounts of nanomaterials increase the viscosity of the membrane casting solution, thereby reducing the phase inversion rate and forming membranes with denser surfaces.^35^ This is because membranes with denser surfaces correspond to a highly favorable environment for foulants to accumulate on the surface. The results of the study clearly show that the PES-ZnO-0.5 membrane had the highest fouling resistance among the nanocomposite membranes.

### 3.4 FTIR Analysis of the Clean Membranes

**Figure 9:**
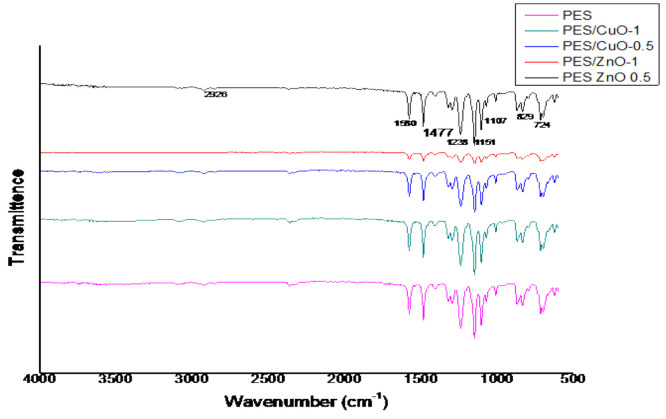
Transmittance peaks of the clean membranes.

Examination of the peaks in the FTIR analysis shows that the bonds associated with PES are prevalent in the all membranes (Figure 9). The transmittance at 829 cm^–1^ in the PES/ZnO nanocomposite indicates the presence of specific chemical bonds and molecular vibrations within the material. This wavenumber corresponds to the stretching vibration of the C–S bond in poly(ether sulfone).^36^ Additionally, the transmittance at 724 cm^–1^ suggests the presence of zinc oxide in the mixture, as this wavenumber corresponds to the characteristic stretching vibration of Zn–O bonds.^36^ These findings are consistent with the analysis of similar materials, where specific wavenumbers were associated with the presence of particular chemical bonds and functional groups.^37^

The transmittance peaks at 1107, 1151, 1238, 1477, 1580, and 2926 cm^–1^ in the PES-ZnO nanocomposite membranes can be attributed to specific molecular vibrations and chemical bonds present in the material. The peak at 1107 cm^–1^ corresponds to the stretching vibration of the C–O bond in poly(ether sulfone).^38^ Additionally, the peaks at 1151 and 1238 cm^–1^ indicate the C–H bending vibrations in the PES-ZnO nanocomposite.^39^ Furthermore, the 1477 and 1580 cm^–1^ peaks are associated with the asymmetric and symmetric stretching vibrations of the C = C bond in the PES-ZnO nanocomposite, respectively.^40^ Finally, the peak at 2926 cm^–1^ represents the C–H stretching vibration, which is characteristic of the PES component in the composite material.^39^

### Surface Porosity of the Clean Membranes

**Figure 10:**
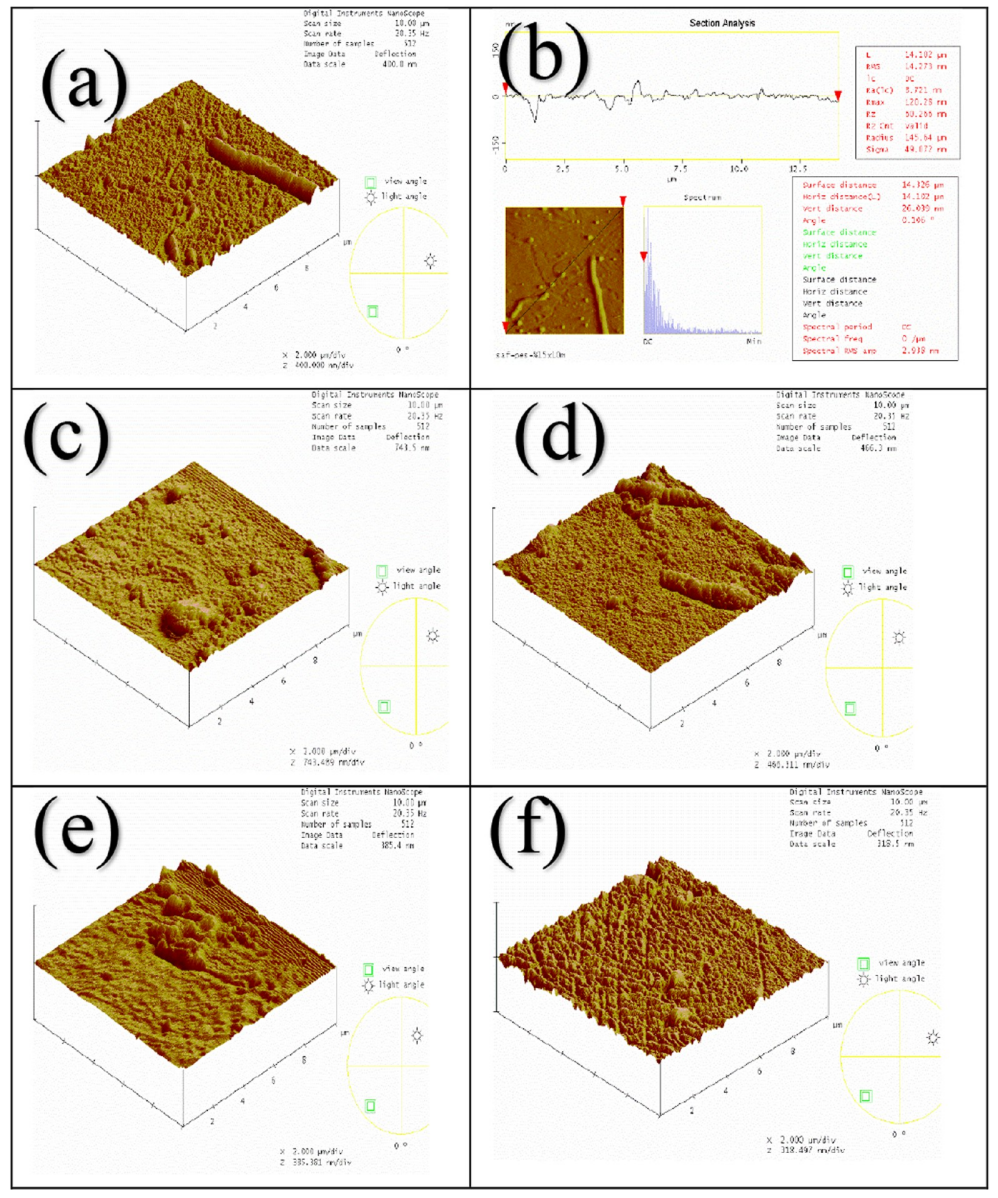
AFM images of the clean membranes (a, b) PES, (c) PES-CuO-0.5, (d) PES-CuO-1, (e) PES-ZnO-0.5, (f) PES-ZnO-1.

Three-dimensional images and surface topographies of the fabricated neat PES (a, b), PES-CuO-0.5 (c), PES-CuO-1 (d), PES-ZnO-0.5 (e), (f) PES-ZnO-1 membranes are taken by AFM device and are shown in Figure 10.

It is observed that the average roughness (Ra) value of CuO and ZnO nanomaterial-doped membranes is slightly lower than that of neat PES polymeric membrane in general, but the Ra value of the membrane containing 1% wt. ZnO is higher than that of neat PES membrane. The membrane with the lowest roughness value is found to be the NC membrane containing 0.5 wt % ZnO and the membrane with the highest roughness value is found to be the NC membrane containing 1% wt. ZnO (Table 3).

**Table 3:** Ra Values of Neat PES, CuO-PES and ZnO-PES Membranes (0–10μm)

Membrane Type	Ra (Average Roughness)
Neat PES	7.573 nm
% 0.5 CuO-PES	7.127 nm
% 1 CuO-PES	7.336 nm
% 0.5 ZnO-PES	6.037 nm
% 1 ZnO-PES	8.452 nm

Assessing the correlation between the wt % CuO content in the membrane and the surface roughness reveals an inverse direction and moderate relationship (r = −0.53). Similarly, evaluating the correlation between the wt % ZnO content in the membrane and the surface roughness reveals a weak relationship in the same direction (r = 0.35).

The average roughness value (Ra) increases in both membranes containing CuO and ZnO with increasing weight percentages of nanomaterial addition to the polymeric membrane. The roughness value of the neat membrane does not decrease with the addition of nanomaterial except for the membrane containing 1% wt. ZnO, which can be attributed to the expansion of nano-ZnO and nano-CuO, which forms the spreading rate of the nonsolvent during the phase transformation precipitation progression and structures a smoother surface. Increasing the concentration of nanomaterial can lead to more nanoparticles accumulating on the membrane surface, which can lead to increased roughness. Therefore, a lower concentration of nanomaterial may cause the membrane surface to be less rough. In addition, a lower concentration of nanomaterial can reduce the roughness by making the membrane more homogeneous. This may allow the membrane to work more smoothly and efficiently.

### 3.6 Mechanical Tests of the Membranes

**Figure 11:**
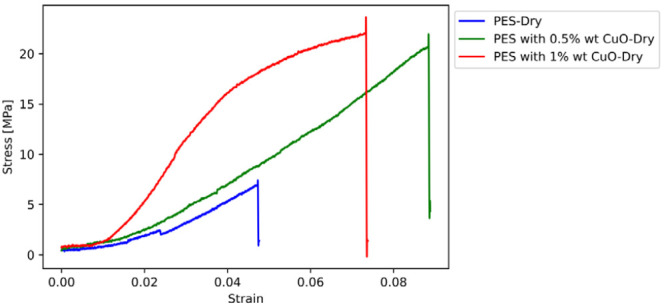
Stress-strain curves of dry PES-based membranes with CuO reinforcement.

**Figure 12:**
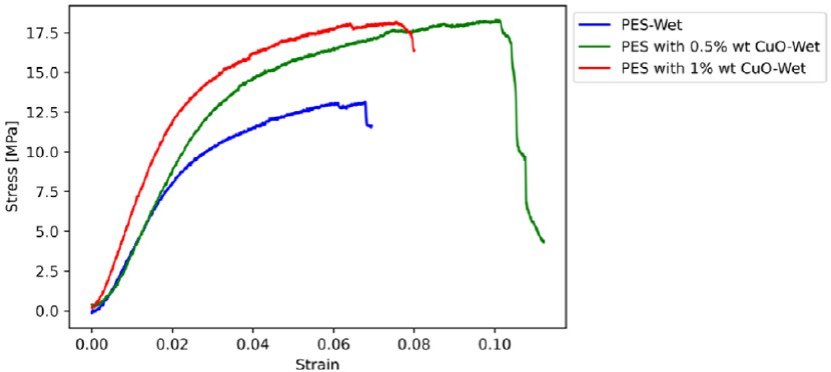
Stress–strain curves of wet PES-based membranes with CuO reinforcement.

**Figure 13:**
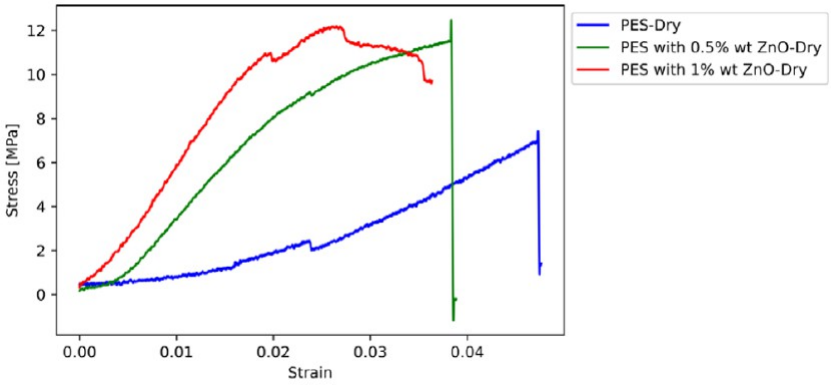
Stress–strain curves of dry PES-based membranes with ZnO reinforcement.

**Figure 14:**
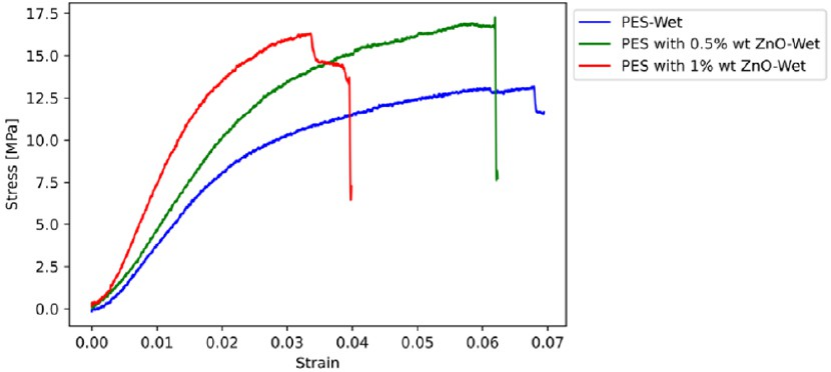
Stress–strain curves of wet PES-based membranes with ZnO reinforcement.

The results of the tensile tests are presented in this section. Figures 11–14 display the stress–strain curves of one sample for each parameter investigated, and Table 4 shows the compiled results that contain modulus of elasticity, and tensile strength and elongation at break.

**Table 4:** Average Mechanical Properties of the Tested Membranes

Sample		Elasticity Modulus [MPa]	Tensile Strength [MPa]	Elongation at Break	Contact Angle [deg]
PES	Dry	17.6	7	0.045	77
	Wet	348.5	12.5	0.07	
PES with 0.5 wt % CuO	Dry	27.8	22	0.09	75
	Wet	377	18.1	0.115	
PES with 1 wt % CuO	Dry	74.8	24	0.075	73
	Wet	604.5	18	0.08	
PES with 0.5 wt % ZnO	Dry	102.3	12.2	0.038	68
	Wet	390.1	17.3	0.062	
PES with 1 wt % ZnO	Dry	383.6	12.25	0.035	65
	Wet	451.8	16.5	0.04	

The stiffness of the membranes is substantially increased by the addition of CuO or ZnO nanoparticles, demonstrating the effect of nanoparticle reinforcement in increasing the mechanical properties, such as stiffness and strength of PES membranes. The transition from dry to wet conditions reveals a correlation between the nanoparticle reinforcement and moisture, leading to significant changes in the mechanical properties. The softening effect observed under wet conditions, as shown by changes in Young’s modulus and tensile strength, can be explained by the hydrophilic nature of the membranes. This is supported by the contact angle measurements, which suggest varying degrees of hydrophilicity among the samples. The decrease in contact angle with increasing nanoparticle content indicates enhanced water absorption, which could explain the variations in mechanical properties in wet conditions. Further investigation of this complex interaction is warranted, possibly through additional characterization, such as water contact angle tests, to clarify the underlying mechanisms involved.

Upon examining the mechanical properties of PES membranes, it is evident that adding CuO or ZnO nanoparticles results in a significant increase in stiffness. These findings demonstrate the clear benefits of nanoparticle reinforcement in enhancing the mechanical properties of PES membranes.

Upon examining the mechanical properties of PES membranes, it is evident that the addition of CuO or ZnO nanoparticles results in a significant increase in stiffness. For instance, the incorporation of 0.5 wt % CuO to dry PES membranes leads to a remarkable (57.95%) increase in the elasticity modulus, from 17.6 to 27.8 MPa. Furthermore, a 1% wt. CuO reinforcement results in an even more impressive increase (324.43%), reaching 74.8 MPa.

This suggests that by carrying a significant portion of the load, nanoparticles lead to a substantial rise in material stiffness. PES membranes reinforced with 0.5 wt % CuO experience a more than 3-fold increase in tensile strength, rising from 7 to 22 MPa. The slightly higher tensile strength of 24 MPa is exhibited by the membranes with 1 wt % CuO reinforcement, indicating diminishing returns at higher nanoparticle concentrations.

The ductility can be better understood by examining the elongation at break. PES membranes with 0.5% wt. nano CuO exhibit a 100% increase in elongation at break compared to nonreinforced PES, indicating improved energy absorption before failure. In wet conditions, the elongation at break for 0.5 wt %nano CuO-reinforced PES increases to 0.115, a 61.7% rise from nonreinforced wet PES, highlighting the intricate interplay between nanoparticle reinforcement and moisture.

The same trend is observed for ZnO nanoparticles. The elasticity modulus of dry PES with 0.5% wt. nano ZnO reinforcement reaches 102.3 MPa, a significant (480.68%) increase from the base PES. Increasing the nano ZnO content to 1% wt. escalates the modulus further to 383.6 MPa, indicating a substantial enhancement in material stiffness. Although the tensile strength sees a more modest increase, the ZnO particles substantially stiffen the matrix, showcasing their effectiveness in enhancing material stiffness.

To fully explain the nature of interfacial bonding in nanoparticle-reinforced polymer matrices, a detailed investigation is required.^41−43^ This may involve molecular dynamics simulations to gain insights into microscale interactions and chemical affinities at the nanoparticle–polymer interface. In the field of polymer nanocomposites, it is widely accepted that the inclusion of nanoparticles has a significant impact on stress distribution within the polymer matrix. This is primarily due to the creation of stress concentrations around the nanoparticles, which act as critical sites for the initiation and propagation of mechanical deformation. In addition, nanoparticles significantly affect the morphological characteristics of the polymer matrix, such as porosity, pore distribution, and dimensions. These changes in the polymer matrix’s microstructural features inevitably affect the composite membranes’ mechanical properties. The relationship between nanoparticle reinforcement and the mechanical properties of the composite is complex, as demonstrated by the interactions between the addition of nanoparticles and the resulting structural changes within the matrix.^44−46^

## 4 Conclusion

In this study, the effects of nano CuO and nano ZnO on the membrane are investigated by comparing the performance of neat PES, PES/CuO, and PES/ZnO membranes produced by the phase inversion method.

With the addition of 0.5% wt. nano CuO and nano ZnO to the neat PES membrane, the pure water flux of the membrane increases by 16.9% and 20.2%, respectively. It is determined that the conductivity, color, total organic carbon, boron, iron, selenium, barium and total chromium removal efficiency of the membrane increased with the addition of 0.5% and 1% wt. nano CuO and nano ZnO to the neat PES membrane. The nanocomposite membranes containing 0.5% wt. nano CuO and nano ZnO exhibit better surface fouling resistance after filtration than the membrane containing 1% wt. nano CuO and nano ZnO. A decrease in pore diameter is observed with the addition of nanomaterial. At the same time, the removal efficiency of contaminants increases. AFM images show that the surface roughness is generally reduced with the addition of nanomaterials and this prevents the attachment of pollutants on the membrane and helps the membrane to operate for a shorter time and with high efficiency. When all the results are evaluated, the nanocomposite PES membrane (PES-ZnO-0.5) doped with 0.5% wt. ZnO is found to be the most suitable membrane for water treatment due to its high flux, high purification performance, and high fouling resistance. The use of nano ZnO in different concentrations by weight can increase the dispersion in the PES polymer. In this way, the decrease in water flux over time can be delayed. This study reveals that the performance of PES membranes, which are widely used in water treatment, can be significantly improved by a low amount of nanomaterial reinforcement.

The mechanical tests’ results indicate that the mechanical properties of PES membranes are determined by the nanoparticle content and type. Higher nanoparticle content generally correlates with increased stiffness and tensile strength but has varying impacts on elongation at break. It is important to consider environmental factors, such as moisture, as they also significantly affect the resultant mechanical properties.
